# Computational Insight into the Intercalating Properties of Cryptolepine

**DOI:** 10.1021/acsomega.4c08666

**Published:** 2025-04-28

**Authors:** George Ferguson, Louie Slocombe, John Lisgarten, David Lisgarten, Colin William Wright, Rosemary Talbert, Rex A. Palmer, Brendan James Howlin, Marco Sacchi

**Affiliations:** † School of Chemistry and Chemical Engineering, University of Surrey, Guildford GU2 7XH, U.K.; ‡ Beyond Center for Fundamental Concepts in Science, Arizona State University, Tempe, Arizona 85287−0506, United States; § Department of Pharmaceutical Chemistry, Faculty of Health Sciences, University of Nairobi, P.O. Box 19676-00202 Nairobi, Kenya; ∥ Biomolecular Research Group, School of Psychology and Life Sciences, Canterbury Christ Church University, North Holmes Road, Canterbury, Kent CT1 1QU, U.K.; ⊥ School of Pharmacy and Medical Sciences (Faculty of Life Sciences), University of Bradford, Richmond Rd, Bradford, West Yorkshire BD7 1DP, U.K.; # Department of Crystallography, Biochemical Sciences, Birkbeck College, Malet St, London WC1E 7HX, U.K.

## Abstract

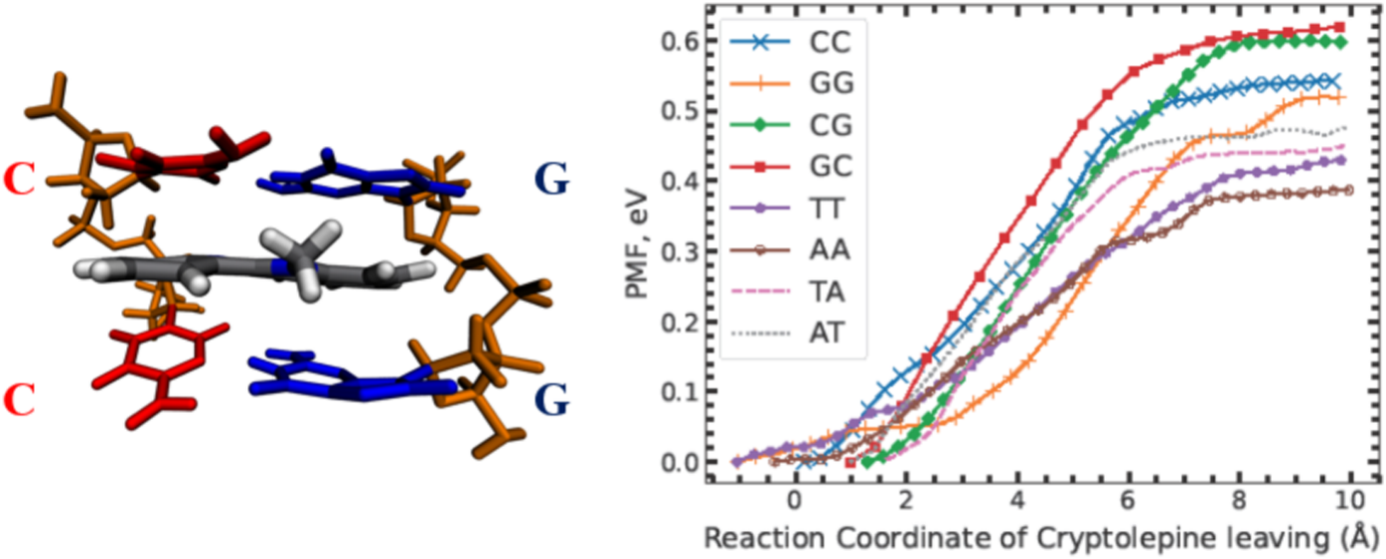

DNA is held together by hydrogen bonding between nucleobases (adenine-thymine, guanine-cytosine) and van der Waals interactions between adjacent base pairs’ π orbitals. Intercalating molecules with quasiplanar structures utilize van der Waals interactions to bind between DNA base pairs. Experimental studies have shown that Cryptolepine preferentially intercalates between nonalternating cytosine and guanine base pairs. However, an atomic-scale mechanism that can explain the selective intercalation is still missing. Using molecular dynamics and density functional theory, we demonstrate how Cryptolepine binds to DNA base pairs, rationalizing its selectivity by analyzing the intermolecular bonding strength predicted by Umbrella Sampling and Free Energy Perturbation calculations. Cryptolepine is stable in all DNA base conformations studied, and the binding is a combination of van der Waals interactions with the nucleobases surrounding its π system and hydrogen bonds with the DNA backbone and nucleobases. Our model predicts a preference for cytosine and guanine base pairs with a more prominent preference for alternating cytosine and guanine base pairs. These findings illustrate Cryptolepine’s binding mechanism to DNA and highlight the importance of hydrogen bonds and van der Waals interactions.

## Introduction

**Figure 1:**
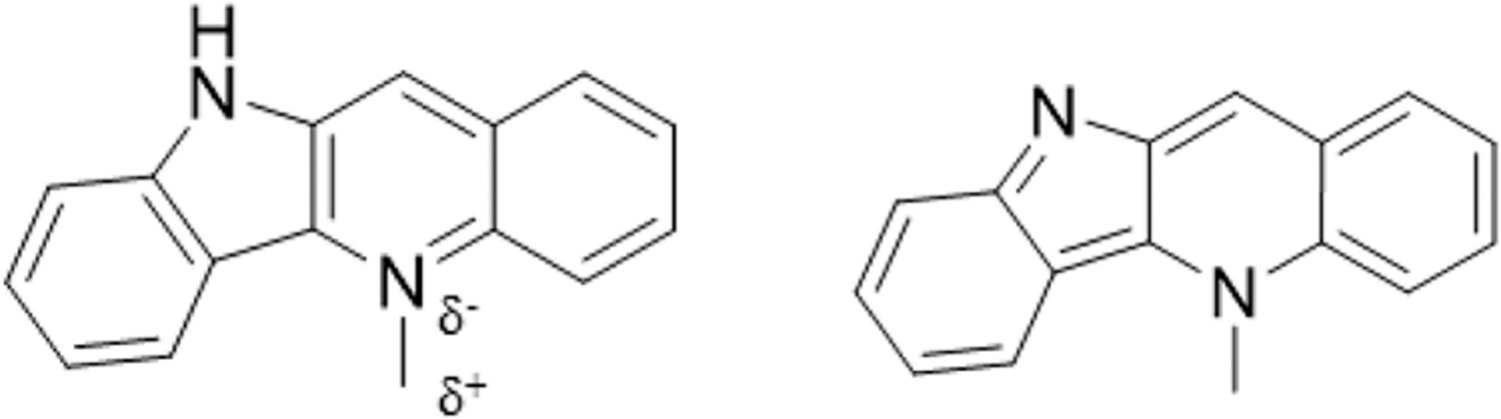
Structure of both charged (left) and neutral (right) Cryptolepine, the δ^–^ and δ^+^, represent charged structures in the molecule as per the force field file. δ^–^ is equal to −0.702*e* and δ^+^ is equal to +0.733*e*.

Cryptolepine is an intercalating drug molecule found in the roots of the *Cryptolepis sanguinolenta* shrub from West Africa.^1^ This herb is widely used in West Africa in traditional medicine for treating malaria and other infectious diseases, and the main alkaloid constituent, Cryptolepine, has been shown to have antimalarial activity^2,3^ among other medicinal properties.^4^ Cryptolepine also possesses cytotoxic properties,^5,6^ which could contribute to its potent antimalarial activity. Figure 1 shows the charged and neutral Cryptolepine structure.

These cytotoxic properties could be due to its ability to intercalate, as cryptolepine inhibits DNA synthesis as well as topoisomerase II enzyme^6,8^ critical to DNA replication. *C. sanguinolenta* may be cultivated, and a ‘green’ process for its extraction and isolation has been developed.^9^ Previous investigations^10^ have also pointed out that Cryptolepine preferentially binds in nonalternating cytosine-guanine chains from data via X-ray structures.^6,9,10^

The intensity of van der Waals interactions between Cryptolepine and DNA is a critical component of the intercalation energy. van der Waals and other noncovalent interactions play a crucial role in the stability of various biological macromolecules such as DNA,^12,13^ where these interactions help hold the double helix macro-structure. By using the same interactions, other similar molecules, such as Quinine,^14^ utilize van der Waals interactions to intercalate^15^ between the base pairs and Ellipticine, which also intercalates^16,17^ and is known to interact with topoisomerase II enzyme, inhibiting it^18,19^ in potentially a similar fashion to Cryptolepine.^6^

Alongside van der Waal interactions, electrostatic and polarization forces play key roles in the interaction energy between the intercalant and nearest nucleobases. The influence of surrounding structures, charged intercalants, and the electronegative atoms holding the DNA nucleobases and located in the backbone provides a surplus of interactions an intercalant can use to bind.^20,21^ The electrostatic interaction can sometimes be more significant than the van der Waals interaction. The oxygen in the sugar backbone and connected to the phosphorus are suitable oxygen acceptors,^20^ which allow molecules such as Cryptolepine to bind via hydrogen bonding easily and, due to its aromatic structure, are polarized to allow this binding. Electronegative structures in the nucleobases above and below an intercalant allow for additional hydrogen acceptor sites to provide further bonding.^21^ In addition, methylated structures similar to Cryptolepine provide additional binding interactions with the major groove surface of guanine and cytosine while exposed due to the electronegative surface of this structure.^21,22^ The methylated structures are similar to Cryptolepine, and we expect this to play a significant role in its stability.

If the cytotoxic properties of Cryptolepine are a product of intercalation, then its selectiveness for nonalternating cytosine-guanine bases could be a useful targeting tool in cancer cell therapy.^6,8,10^ Various successful antitumor drugs demonstrate intercalating properties,^19^ and Cryptolepine has a very similar structure to anticancer agents^16^ with studies investigating its use;^6^ thus, the detailed knowledge of the π-π interactions could prove crucial in furthering the understanding of the molecule and its potential role as an antitumor agent.

The X-ray structures of Cryptolepine reported by Lisgarten et al.^10^ show four molecules of Cryptolepine intercalated into a B-DNA structure between C-G base pairs with an A-T base pair in between the two intercalation sites. The X-ray structure also features two capping Cryptolepine molecules at the ends of the DNA. This experimental structure forms the basis and starting point of the present computational investigations, which aim to investigate how the structure is holding itself together and gain an estimation of the strength of the interaction. We also aim to explain the nature of the selectivity predicted by the experimental results.

## Methodology

**Figure 2:**
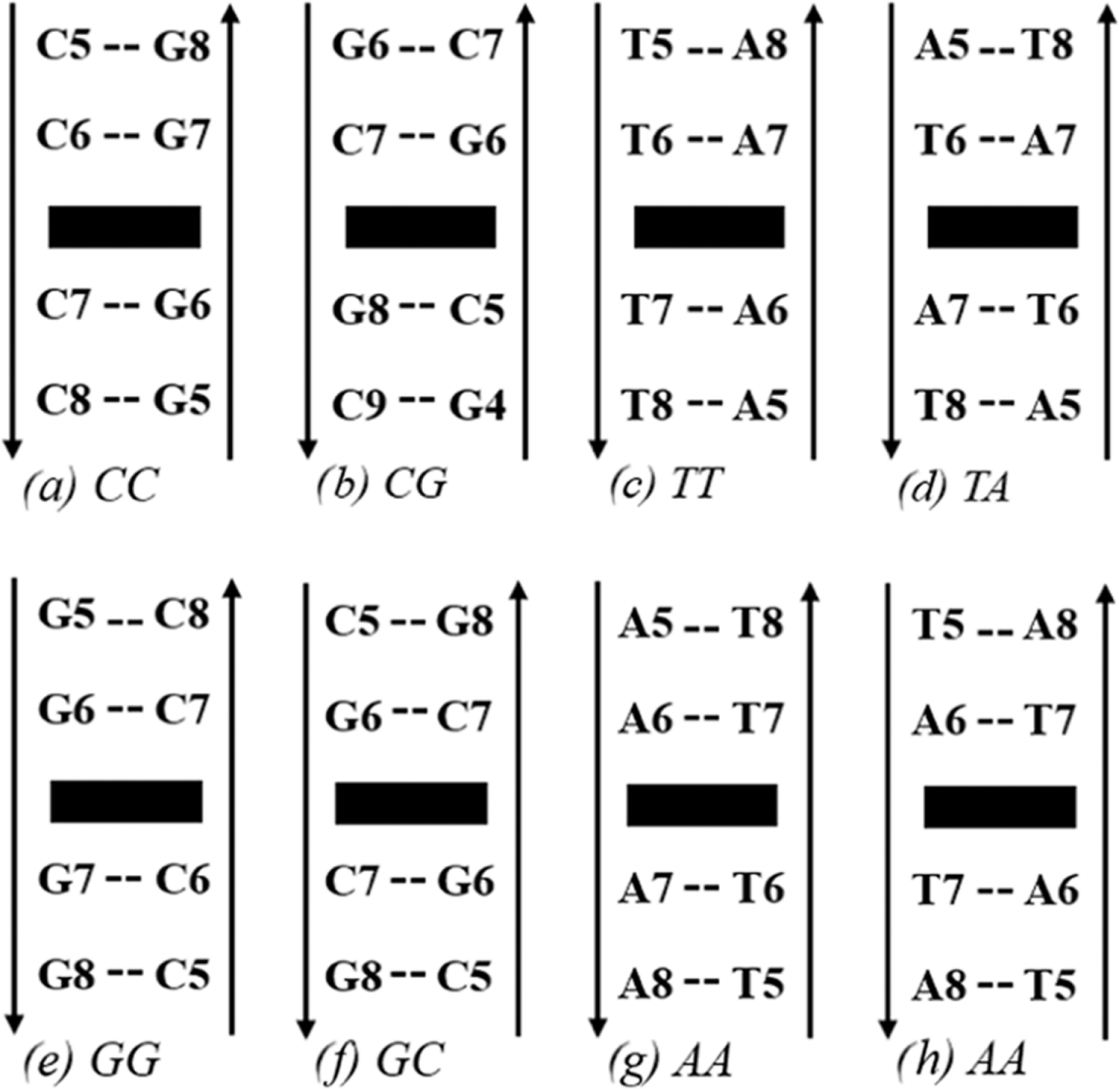
Sequence schematic for the four nearest base pairs to Cryptolepine (presented as a black rectangle). These sequences are repeated above and below the outlined figure to create the DNA chain we use to simulate.

Classical MD simulations were performed via GROMACS 2021.1.^23^ The MD trajectories were set up with a helix structure of 12 B-DNA base pairs built in the Avogadro software.^24^ The sequence of DNA bases is defined by the identity of the left DNA nucleobase above and below the left single hexagon ring of Cryptolepine while facing the major groove of DNA. It is described as such: CC (cytosine, cytosine), CG (cytosine, guanine), GC (guanine, cytosine), GG (guanine, guanine), TT (thymine, thymine), TA (thymine, adenine), AT (adenine, thymine), and AA (adenine, adenine) (See Figure 2 for more details). Initially, the Molecular Operating Environment (MOE) software^25^ was used to quickly optimize the DNA-Cryptolepine system via a classical AMBER 10 force field.^26^ This optimization allowed the DNA base pairs to displace, creating space for the intercalant without disrupting the overall structure of the DNA. This was then prepared for GROMACS. All structures consider only a single protonated Cryptolepine molecule intercalated into the DNA structure (see charged structure in Figure 1).

The protonated Cryptolepine was parametrized using the CHARMM GUI with the ligand reader^27,28^ to create force field parameters for use with the CHARMM 36 force field^29^ via GROMACS.^23^ MD calculations at a constant number of atoms, volume, and temperature (NVT ensemble) were performed on previously mentioned systems to investigate the molecule’s stability while equilibrating at human body temperature (310.15 K). Solvent effects were accounted for through an explicit solvent model via the TIP3P^30^ force field. The protonated form has a p*K*_a_ of 11.2,^31^ implying it will remain protonated in a water solvent.

For Umbrella Sampling (US) calculations,^32^ steered MD force parameters were applied to the Cryptolepine molecule to remove the ligand from the DNA structure. Cryptolepine was pulled via its Center of Mass (CoM) with reference to the surrounding DNA nucleobase CoM. The reaction path produced via this pulling was used to perform an Umbrella sampling across it and generate a PMF graph. Sampling was performed for all eight base pair combinations tested with a step size of one fs over five nanoseconds.

Finally, to complement the US calculations, Free Energy Perturbation (FEP) calculations^33^ were performed, decoupling the Cryptolepine molecule in solvent and when bound to DNA. Charge and van der Waals interactions were turned off gradually, and the system was equilibrated before running the molecular dynamics. The simulation ran over one ns in all cases, and the final result was the drug free in the solvent against the drug binding in the system. Three separate calculations were performed on each system, and the average binding energy value between these three was taken as the final binding energy. The number of steps varied depending on the structure, with 41 steps for Cryptolepine in a solvent; 45 steps for CC, CG, GC, TT, TA, AT, and AA; and 51 steps for GG. GG was less stable than the other seven nearest neighbors, and instead, we only took the output of one FEP calculation for the value. For a minimized structure, we used the energy-minimized structures from the input structures for FEP to compare to the QM (see Table 2). The eight structures were converged to 100 kJ mol^–1^ nm^–1^.

As part of our analysis, we perform a Boltzmann probability distribution to describe the occupancy of all the binding energies we have predicted for Cryptolepine (see eq 1 in the SI for more information). For an experimental estimate, we have used Figure 2 from Lisgarten et al.^10^ to provide an estimate toward the experimental occupancy as it represents the micromolar mass of Cryptolepine between the listed DNA sequences, which we have taken to correlate to binding affinity. The figure is based on results from Ren and Chaires^34^ utilizing the “Rend and Chaires competition dialysis method.” The estimated values are shown in Figure 6.

To complement the MD calculations, we also run a Quantum Mechanical (QM) Density Functional Theory (DFT) calculation to investigate the energy contribution toward the binding of Cryptolepine to the surrounding DNA via an Energy Decomposition Analysis (EDA) using the Absolutely Localized Molecular Orbitals (ALMO) method.^35^ We initially perform an optimization via NWChem 7.2.2^36^ on a ring model DNA structure of all eight base pair sequences surrounding the intercalant before running the EDA calculation with a single potion (SP) calculation in the Qchem 6.1 software.^37^ The calculations were run with a BLYP exchange-correlation (XC) functional^38^ along with a 6–31+G* basis set and a GD3 dispersion correction.^39^ Force convergence was set to 0.05 eV of Å^–1^. A COSMO implicit solvent^40^ was used in the optimization script, but COSMO could not be used in the EDA, and instead, SMD^41^ was used to represent the implicit solvent in Qchem. The energy contribution is then described as a percentage contribution toward the structure’s overall energy. For further details on the QM model and figure, see SI and figure S1.

## Results and Discussion

### Molecular Dynamics Investigation into Intercalating Stability

We can investigate if the system can remain stable between DNA in a dynamical simulation, determining whether the intercalant can disrupt the DNA structure or if it will slip outside the DNA while equilibrating at 310.15 K.

Although Cryptolepine is more unstable in AT and TA, the RMSD values are still small, given the molecule’s and system’s size. Thus, we can still conclude that classically the molecule remains stable in all cases when intercalated. For all cases except AT and TA, we see no significant difference between base pairs; therefore, no preference can be detected. For the stability of the DNA chains, see Table S1 in the SI.

During the calculation, Cryptolepine shifts around the DNA gap, moving between the DNA backbones but remaining stable inside the DNA. During the 20 ns simulation, we saw that Cryptolepine can shift significantly, rotating perpendicular to the nearest DNA pairs. This shift either rotates back to its original position, exposing the methylated structure to the major groove of the DNA, or rarely fully rotates to expose the methylated structure on the minor groove (Table 1).

**Table 1:** RMSD Average and Standard Deviation from MD Calculation of a Single Cryptolepine Molecule Intercalated Inside the Center of a 12 Base Pair DNA Chain in the Solvent Over 20 ns NVT at 310.15 K^a^

DNA bases	Average RMSD	STD
CC	0.265	0.0117
CG	0.264	0.0157
GC	0.282	0.0145
GG	0.336	0.0186
TT	0.278	0.0167
TA	0.302	0.0333
AT	0.408	0.0627
AA	0.283	0.0175

a We see consistent stability in all cases except for TA and AT. All distance values are in Å, and each MD ran for 20 ns (See Figure S2 in the SI for more details on the RMSD calculation).

### Observation of Hydrogen Bonding Playing a Key Role

We have noticed via MD simulations that Cryptolepine binds tightly not just by π–π interactions but also by forming a series of hydrogen bonds along the outside of the aromatic ring structure and the electronegative oxygen atoms present in the DNA backbone. We see an indication that it plays a crucial role in the stability of the intercalant while intercalated.

Several protons across Cryptolepine happen to be positioned where they could play a key role in binding: the methylated carbon structure and planar hydrogens along the outside of the Cryptolepine structure. The methylated carbon structure coincides with the DNA base pair gap, allowing it to form hydrogen bonds with the nucleobase pair acceptor atoms. These hydrogen bonds often fall below 3.0 Å, indicating regular hydrogen bond formation despite the dynamics of the structure. This is an interaction encountered by other methylated intercalants and is expected to occur in this structure.^21,22,42,43^ The methylated structure is conveniently positioned on Cryptolepine during intercalation to interact directly with the nucleobase pair gap.

We also see evidence that hydrogen atoms connected to the planar ring of Cryptolepine have weak interactions with oxygen atoms at the DNA base gap. This may cause slight bending between the DNA bases toward Cryptolepine as this hydrogen attracts the ends of the DNA bases. The distances here are on the limits of hydrogen bonding, often sitting at 3.0 Å apart and forming sporadically before moving apart. While this interaction is likely very weak, the -NH group is better positioned to interact with the electronegative hydrogen acceptors at the DNA base gap. We often see in simulations that the –NH hydrogen is angled toward nucleobases, which have an electronegative atom, typically oxygen, directly above or below. The nucleobases thymine and cytosine both have oxygen atoms positioned where they can interact with the -NH group and form hydrogen bonds. These hydrogen bonds, however, are limited via the small angle between the donor and acceptor atoms, and stronger bonds may instead form via backbone connection rather than directly to the nucleobase.

**Figure 3:**
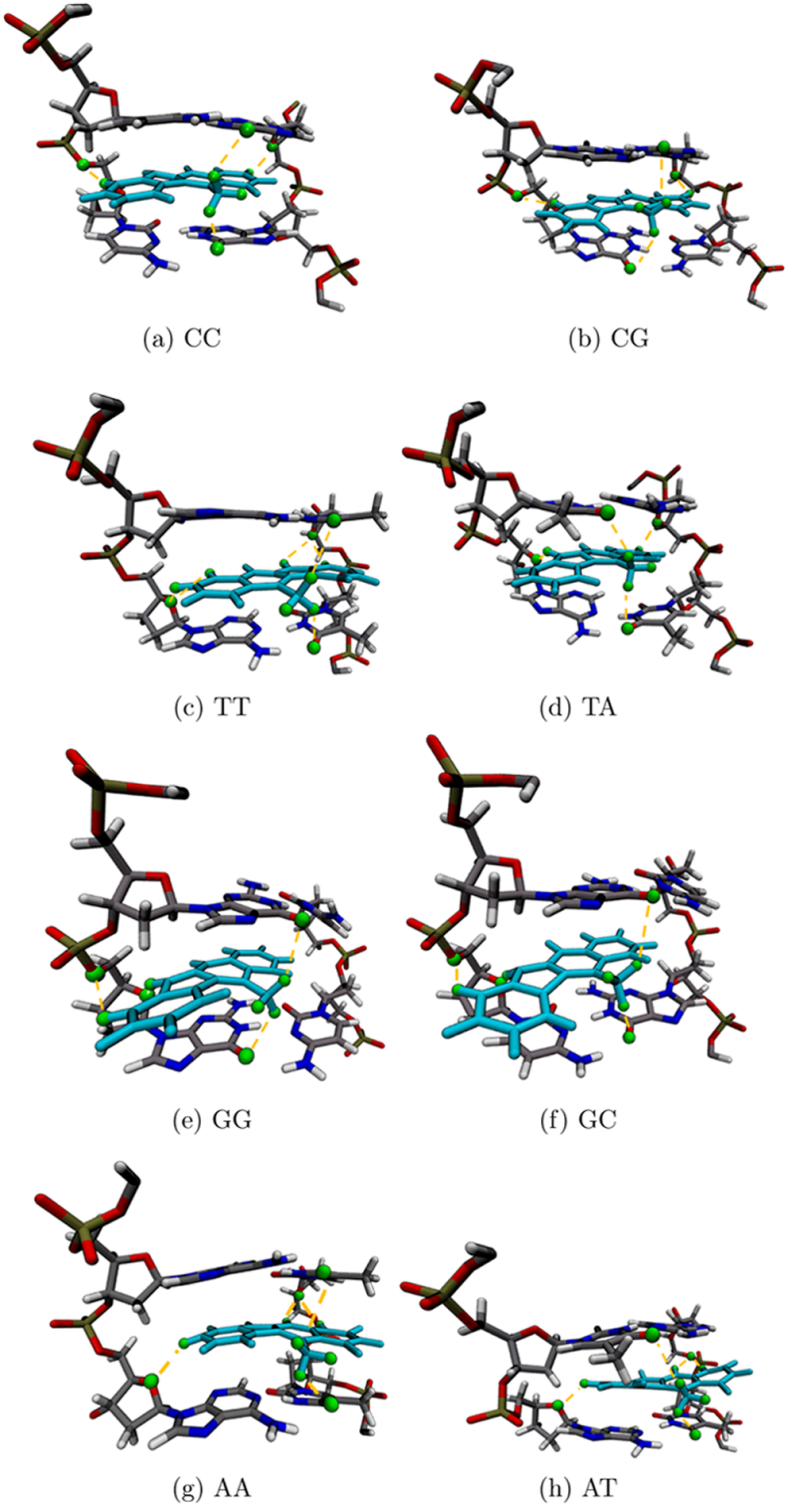
Snapshots of all eight simulated structures from RMSD calculations. The left side (a–f) represents all C-G base pairs with Cryptolepine (teal) intercalated. The right side (c, d, g, h) represents all A-T base pairs with Cryptolepine intercalated. Highlighted green are atoms that will hydrogen bond, helping hold Cryptolepine in place.

We also see hydrogen bonding from the Cryptolepine onto the DNA backbone due to the electronegative oxygens in the sugar and phosphorus backbone. Various intercalants interact in this fashion, helping bind the drug to the DNA in an alternative method to van der Waals binding.^20−22,44,45^ We see through the MD dynamics that the Cryptolepine molecule will often shift between each backbone, forming hydrogen bonds with one side before separating and reforming new bonds on the alternative backbone during the dynamics. Sporadic hydrogen bond formation occurs regularly across the structure as the drug shifts during dynamics, and the most common hydrogen bonds are shown in green in Figure 3. We also notice that, on average, hydrogen bonds are formed further away in A-T base pairs compared with C-G base pairs. However, we do not see a clear correlation between hydrogen bonding and base pair preference. A common section for hydrogen bonding is located at the sugar backbone with the electronegative oxygen atom and the nearby phosphorus structure (See SI Tables S2–S17 for a list of hydrogen bonds).

However, given the dynamic nature of MD, hydrogen bond investigation is limited as part of the dynamics, which results in the variability of the geometry and thus shifting of the hydrogen bond distance and angle across the simulation. Although the MD can calculate the influence of hydrogen bonds,^29,46−48^ the sporadic nature and dynamics of the calculation can result in a lack of accuracy in approximating the hydrogen bonds fully. To accommodate this, we have run *ab initio* methods to compare them to an optimized MM structure. We optimized DNA ring structures surrounding the intercalant and compared the predicted hydrogen bond donors and acceptors to MM optimization.

From Table 2, the average comparison between QM and MM hydrogen bonds is around a similar value. Both hydrogen bonds and angles from the MM are similar to QM values but are often further apart. The overall increase in bond length between QM and MM is likely due to the difference in convergence parameters, as QM was more tightly optimized. In contrast, the MM optimized the entire DNA chain and the surrounding explicit solvent to a lower convergence.

**Table 2:** List of the Average Hydrogen Bonds of the Nearest Neighboring Nucleobase Pair to Cryptolepine, Listing Both the QM and MM Values^a^

DNA bases	Av. H-Bond (QM) (Å)	Av. H-Bond angle (QM) (deg)	Av. H-Bond (MM) (Å)	Av. H-Bond angle (MM) (deg)
CC	2.722	127.14	2.815	129.17
CG	2.668	133.40	2.708	132.63
GC	2.816	134.05	2.836	123.56
GG	2.742	137.50	2.677	132.87
TT	2.546	133.62	2.728	138.22
TA	2.630	134.38	2.656	138.97
AT	2.820	135.23	2.606	137.74
AA	2.664	139.78	2.819	136.38

a We define the occurrence of the hydrogen bond to be below 3.3 Å in distance and between 90 and 270 deg for the hydrogen bond angle.

We also perform an EDA calculation to further our QM investigation and break down the energy contribution toward the intercalant. Utilizing EDA calculations, we have found the total contribution of energy toward the total binding energy.

Where, in the above figure, the electrostatic is the contribution toward the attractive electrostatic interaction between Cryptolepine and the nearest DNA bases, Pauli represents the Pauli repulsion due to occupied electron orbitals, dispersion represents the long-range van der Waal and π-π stacking attractive interactions, and finally the Polarization and CT (Charge Transfer) values are in the orbital contributions to the energy. The Electrostatic and Pauli Repulsion terms also include the addition of an implicit solvent as part of their contribution.

**Figure 4:**
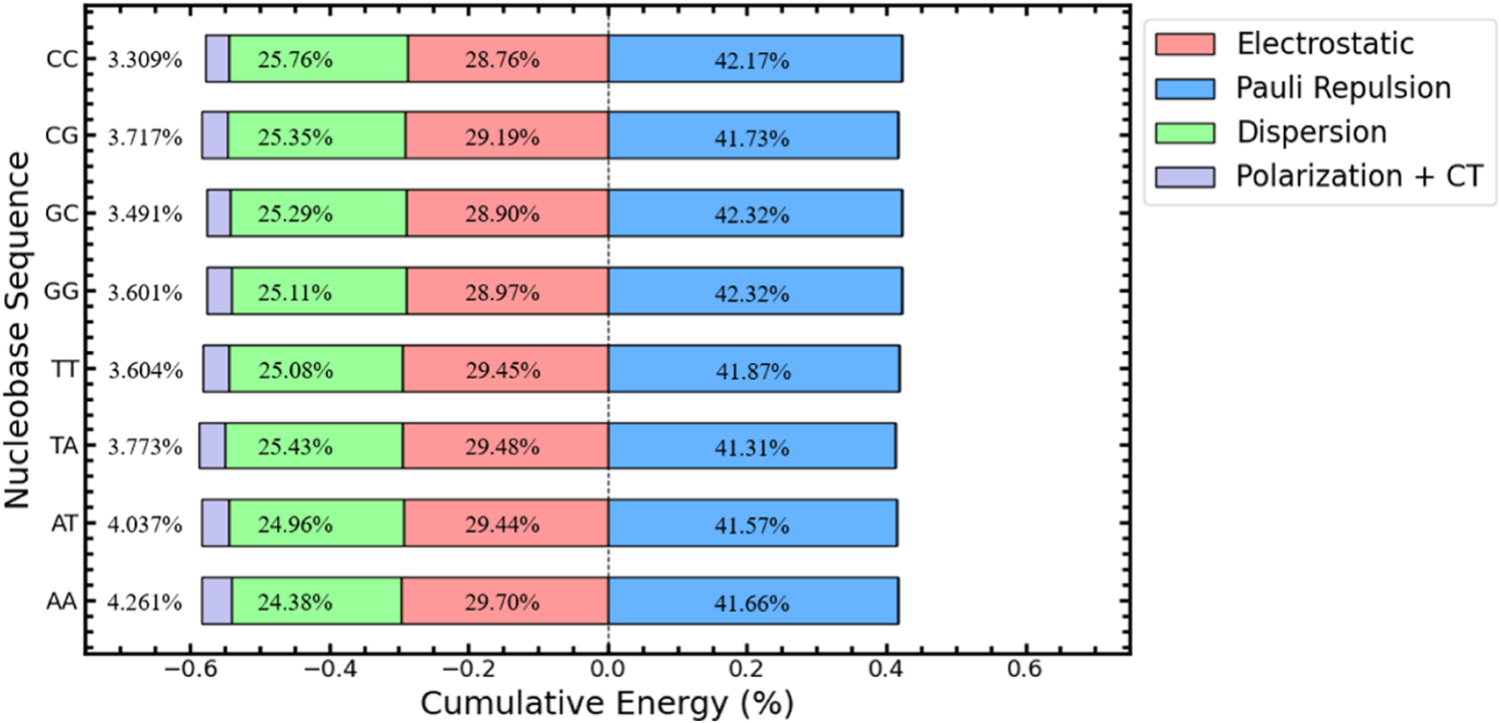
EDA Graph of all DNA sequences with a ring model DNA structure around Cryptolepine. Contributions to energy are listed as percentages of their total contribution to the magnitude of the interaction energy.

The EDA calculations listed in Figure 4 demonstrate the cumulative contribution toward the interaction energy for Cryptolepine while intercalated. We see a significant contribution from the electrostatic interaction ranging from 28.76 to 29.70% for CC and AA, respectively. The dispersion contributes significantly toward the interaction energy with a contribution of 24.38 to 25.76% for AA and CC, respectively. Together, these can overcome the repulsive contributions from the Pauli interactions and bind the intercalant to the DNA. Polarization and charge transfer terms contribute further, binding the intercalant tighter to the structure. We do not see a significant difference between the structures in the QM picture.

### Umbrella Sampling Calculations to Determine a Binding Energy Estimate

The molecule’s stability does not reveal any particular clear preference for any nearest neighbor. To investigate further, we performed steered MD calculations and ran US calculations across the reaction coordinate provided by the steered MD to estimate the binding energy of Cryptolepine while intercalated.

**Figure 5:**
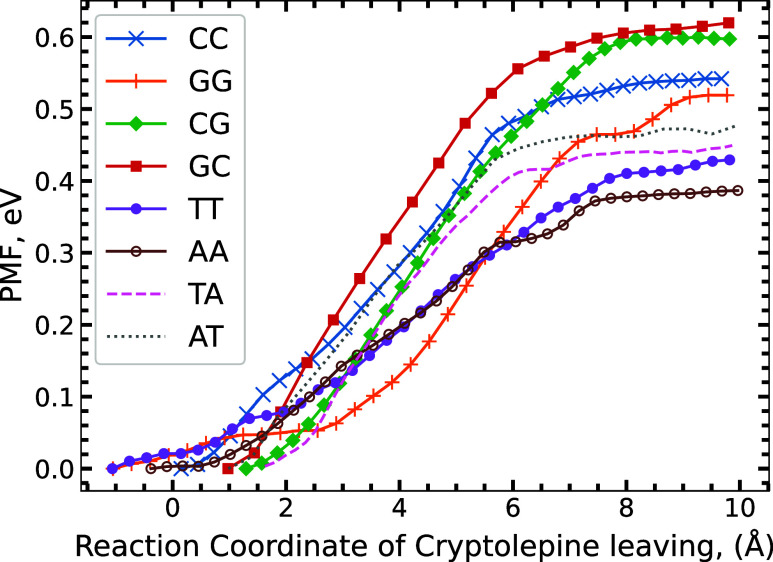
Potential of Mean Force (PMF) graph over the reaction coordinate as Cryptolepine leaves the DNA, leaving behind a hole over all eight systems with the bottom of their potential well set to zero.

In all eight systems we tested, we see a clear thermal preference toward C-G base pairs in Figure 5, specifically nonalternating C-G sequences (CC and GG). CG and GC base pairing achieve the largest dissociation energy, respectively, placing them as the strongest pairing for Cryptolepine to bind between. We see a sequence preference following CG and GC, CC and GG, TA and AT, and TT and AA. This provides a similar preference to the experimental population prediction,^10^ except for the flip between the CC and GG and CG and GC preference. We believe the tighter binding of alternating DNA sequences results in a stronger binding energy for the intercalant between the molecules, providing a preference toward CG and GC and TA and AT over their nonalternating counterparts. Our results show a clear preference for C-G base pairs thermally, which supports experimental evidence, and A-T base pairs match the experimental trend.

We also see in Figure 5 that the binding energy varies when comparing the tightest binding to the weakest binding with AA having the smallest dissociation energy. Our results show that despite the range in dissociation energy, Cryptolepine remains stable in all DNA base pairs we tested and is tightly bound. Unfortunately, the US does not provide a reverse barrier to entry in our test, leaving kinetic selection out.

### Further Binding Energy Investigations via Free Energy Perturbation Calculations

In addition to US calculations, we also performed FEP calculations on the system. We slowly decoupled the Cryptolepine molecule via charge and van der Waals interactions to estimate the binding energy.

The FEP binding energies provide Δ*G* values similar to those of the US, overall being slightly tighter than predicted, as seen in Table 3. We reason that the FEP provides a more accurate estimate of the true binding energy of Cryptolepine in each system due to the lack of pull force biases provided by the US. Considering this, we see a more complicated picture of the binding energy. Although the largest binding between the base pairs remains the nonalternating GC base pair, the CC and GG binding energies have increased beyond CG, as seen in Table 3. The methylated component helps to bind the intercalant to the structure. The electronegative surface of C-G pairs provides many binding sites for a C–H bond to connect. The methylated hydrogen bond is known to assist in the stability of intercalants depending on the number of binding sites,^42^ and its position at the center allows it to easily interact in the major groove with the surrounding electronegative surface and electronegative oxygen and nitrogen.^21^ The methylated structure is positioned to interact across the surface of guanine in the GC structure. It cannot easily interact with the guanine in the CG structure, helping to explain the preference for GC over CG. However, the difference is more significant than we would expect despite this. The electronegative major groove surface of C-G base pairs describes the attractive preference toward C-G base pairs over A-T.

**Table 3:** FEP Gibbs Free Energy (Δ*G* eV) for Each Tested Intercalant-Base Pair Interaction^a^

DNA bases	Δ*G*_FEP_	Δ*G*_FEP_ error	Δ*G*_US_	Δ*G*_FEP_ – Δ*G*_US_
CC	–0.5756	±0.029	–0.54	–0.04
CG	–0.5338	±0.023	–0.60	+0.07
GC	–0.6571	±0.032	–0.62	–0.04
GG	–0.5552	±0.019	–0.52	–0.04
TT	–0.5076	±0.029	–0.43	–0.08
TA	–0.4929	±0.021	–0.45	–0.04
AT	–0.5257	±0.020	–0.49	–0.04
AA	–0.4722	±0.020	–0.38	–0.09

a In addition, the binding energy estimates (Δ*G*, eV) of the US for each equivalent base pair.

The FEP continues the trend set by the US, demonstrating a clear preference for C-G site binding, although favoring the alternating sequences instead of nonalternating. The tight binding pocket produced by the Cryptolepine finds multiple ways to bind to the surrounding structure via hydrogen bonding and π–π interactions. We believe that C-G bases bind tighter than A-T bases due to more potential stacking interactions via polarized hydrogens, which will be attracted via electrostatic interactions to the negative π electron potentials from the aromatic ring structures. The binding is tighter, as C-G bases have more hydrogen acceptors available in the major groove. The other reason we find alternating sequences bind tighter is that they form tighter twist-angle DNA strands than nonalternating sequences. This extra binding will also apply to the intercalant when placed inside, causing additional binding energy and resulting from the overall DNA structure.

Compared to other structures in the literature, our reported binding energies follow similar values to other intercalants, which have been simulated via MD techniques.^15^ For Quinine, a binding energy of 0.516 and 0.507 eV for A-T and C-G base pairs is found respectively.^15^ Our intercalant has a broader range of binding energies, as seen in Table 3, but this could be due to investigating various base pair combinations rather than two. The binding preference for Quinine is seen to favor a minor groove binding, while we investigate major groove binding. We believe this is why they predict the A-T base pair preference, while we expect a C-G binding preference.

## Probability of Occupation

Finally, we can investigate the probability that any possible state would be filled by Cryptolepine, as predicted by the potential energy differences in Table 3. Using a Boltzmann probability distribution, we can investigate the thermodynamical occupation at equilibrium.

**Figure 6:**
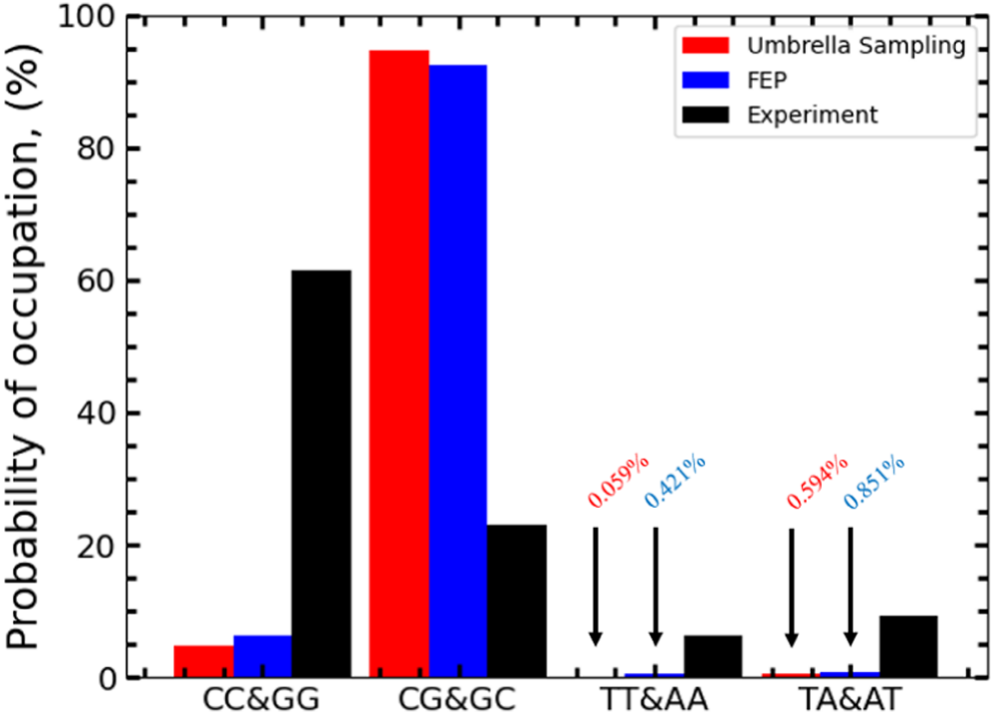
Occupation Probabilities for Cryptolepine intercalated between base pairs. The occupation probability was calculated via a Boltzmann probability distribution, starting with the US binding energy (red), FEP binding energy (blue), and finally, an estimation of experimental predictions (black).

The thermodynamic occupation comparison (Figure 6) shows a clear difference from the exact prediction of the experimental results. Still, it does support the general expectation of a favored C-G base pair state. However, our results show a clear bias toward the alternating C-G sequences with a predicted percentage occupancy of 94.7% in the US binding and 92.5% in the FEP binding compared to all other tested states. We find little occupation in A-T sequences, with a small percentage occupation of 0.595% in the US binding and 0.851% in the FEP binding for alternating A-T sequences. Nonalternating A-T sequences have an occupation probability of 0.059% in US binding and 0.421% in FEP binding. Finally, the C-G nonalternating sequence had a probability of occupation of 4.685% in the US and 6.273% in the FEP binding. Both results show a clear thermal preference for the C-G base pairs, especially toward nonalternating sequences.

## Conclusions

Our MD results demonstrate that Cryptolepine forms a stable intercalated structure with all eight tested DNA sequences in a B-DNA structure at a human body temperature (310.15 K). We find little indication of nucleobase site preference from the RMSD, but instead, we see a stable intercalation site throughout the simulation for all structures. Further investigations to find binding energy estimates have found tight and varying binding energies depending on the DNA sequence. The EDA analysis suggests that the binding of Cryptolepine in our system is mainly driven by a combination of electrostatic attraction and dispersion. US calculations have determined a patterned preference, suggesting a strong thermodynamic preference for sequences of C-G DNA nucleobases with a significant preference for alternating sequences, which goes against experimental predictions. This was then backed up by FEP calculations, which predicted very similar patterns and occupation.

The preference for non-alternating sequences seen in experimental results could arise from methodological factors. Alternatively, the MD force field could be inaccurate in estimating Cryptolepine's interactions with its surroundings. Furthermore, our simulations might be insufficient to fully represent the various interactions this intercalant engages in with different DNA sequences. Despite this, our results agree with the overall sequence preference toward C-G bases, which we speculate is due to a stronger electrostatic interaction with C-G bases than A-T bases when binding via π stacking.

## Data Availability

In addition to further details provided in the Supporting Information, input, parameter, and analysis files sufficient to reproduce the results published in this work are available on GitHub at https://github.com/GeorgeFergie/Cryptolepine_Intercalation_Paper. Additional raw data used and analyzed during the study are available from the corresponding author upon request.

## Supporting Information Available

The supplementary information file contains a description of how the QM structures were generated, the equation used to calculate the Boltzmann probability values in Figure 6, RMSD plot of Table 1, and RMSD table of DNA chains in solvent to test stability and a list of all hydrogen bonds to nearest acceptor atoms for Cryptolepine to bind to in the MM and QM optimization (PDF)

## References

[ref1] Wright C. W.; Addae-Kyereme J.; Breen A. G.; Brown J. E.; Cox M. F.; Croft S. L.; Gökçek Y.; Kendrick H.; Phillips R. M.; Pollet P. L. Synthesis and evaluation of cryptolepine analogues for their potential as new antimalarial agents. J. Med. Chem. 2001, 44, 3187–3194. 10.1021/jm010929+. 11543688

[ref2] Grellier P.; Ramiaramanana L.; Millerioux V.; Deharo E.; Schrevel J.; Frappier F.; Trigalo F.; Bodo B.; Pousset J. Antimalarial activity of cryptolepine and isocryptolepine, alkaloids isolated from Cryptolepis sanguinolenta. Phytother. Res. 1996, 10, 317–321. 10.1002/(SICI)1099-1573(199606)10:4<317::AID-PTR858>3.0.CO;2-0.

[ref3] Wright C.; Phillipson J.; Awe S.; Kirby G.; Warhurst D.; Quetin-Leclercq; L A. Antimalarial activity of cryptolepine and some other anhydronium bases. Phytother. Res. 1996, 10, 361–363. 10.1002/(SICI)1099-1573(199606)10:43.0.CO;2-N.

[ref4] Olajide O. A.; Ajayi A. M.; Wright C. W. Anti-inflammatory properties of cryptolepine. Phytother. Res. 2009, 23, 1421–1425. 10.1002/ptr.2794. 19288476

[ref5] Laryea D.; Isaksson A.; Wright C. W.; Larsson R.; Nygren P. Characterization of the cytotoxic activity of the indoloquinoline alkaloid cryptolepine in human tumour cell lines and primary cultures of tumour cells from patients. Invest. New Drugs 2009, 27, 402–411. 10.1007/s10637-008-9185-5. 18853102

[ref6] Bonjean K.; De Pauw-Gillet M.-C.; Defresne M.-P.; Colson P.; Houssier C.; Dassonneville L.; Bailly C.; Greimers R.; Wright C.; Quetin-Leclercq J.; et al. The DNA intercalating alkaloid cryptolepine interferes with topoisomerase II and inhibits primarily DNA synthesis in B16 melanoma cells. Biochemistry 1998, 37, 5136–5146. 10.1021/bi972927q. 9548744

[ref8] Dassonneville L.; Lansiaux A.; Wattelet A.; Wattez N.; Mahieu C.; Van Miert S.; Pieters L.; Bailly C. Cytotoxicity and cell cycle effects of the plant alkaloids cryptolepine and neocryptolepine: relation to drug-induced apoptosis. Eur. J. Pharmacol. 2000, 409, 9–18. 10.1016/S0014-2999(00)00805-0. 11099695

[ref9] Abacha Y.; Forkuo A.; Gbedema S.; Mittal N.; Ottilie S.; Rocamora F.; Winzeler E.; van Schalkwyk D.; Kelly J.; Taylor M.; et al. Semi-synthetic analogues of cryptolepine as a potential source of sustainable drugs for the treatment of malaria Human African Trypanosomiasis and Cancer. Front. Pharmacol. 2022, 13, 875647 10.3389/fphar.2022.875647. 35600849 PMC9119314

[ref10] Lisgarten J. N.; Coll M.; Portugal J.; Wright C. W.; Aymami J. The antimalarial and cytotoxic drug cryptolepine intercalates into DNA at cytosine-cytosine sites. 2002; Vol. 9. 10.1038/nsb729 11731803

[ref12] Friedman R. A.; Honig B. The electrostatic contribution to DNA base-stacking interactions. Biopolymers 1992, 32, 145–159. 10.1002/bip.360320205. 1637989

[ref13] Hobza P.; Sponer J.; Polasek M. H-Bonded and Stacked DNA Base Pairs: Cytosine Dimer. An Ab Initio Second-Order Moeller-Plesset Study. J. Am. Chem. Soc. 1995, 117, 792–798. 10.1021/ja00107a023.

[ref14] Achan J.; Talisuna A. O.; Erhart A.; Yeka A.; Tibenderana J. K.; Baliraine F. N.; Rosenthal P. J.; D’Alessandro U. Quinine, an old anti-malarial drug in a modern world: role in the treatment of malaria. Malar. J. 2011, 10, 144 10.1186/1475-2875-10-144. 21609473 PMC3121651

[ref15] Punihaole D.; Workman R. J.; Upadhyay S.; Van Bruggen C.; Schmitz A. J.; Reineke T. M.; Frontiera R. R. New Insights into Quinine-DNA Binding Using Raman Spectroscopy and Molecular Dynamics Simulations. J. Phys. Chem. B 2018, 122, 9840–9851. 10.1021/acs.jpcb.8b05795. 30336027 PMC6425490

[ref16] Lin I.-C.; von Lilienfeld O. A.; Coutinho-Neto M. D.; Tavernelli I.; Rothlisberger U. Predicting Noncovalent Interactions between Aromatic Biomolecules with London-Dispersion-Corrected DFT. J. Phys. Chem. B 2007, 111, 14346–14354. 10.1021/jp0750102. 18052270

[ref17] Li S.; Cooper V. R.; Thonhauser T.; Lundqvist B. I.; Langreth D. C. Stacking interactions and DNA intercalation. J. Phys. Chem. B 2009, 113, 11166–11172. 10.1021/jp905765c. 19719266

[ref18] Froelich-Ammon S. J.; Patchan M. W.; Osheroff N.; Thompson R. B. Topoisomerase II Binds to Ellipticine in the Absence or Presence of DNA.: Characterization of Enzyme Drug Interactions by Fluorescence Spectroscopy. J. Biol. Chem. 1995, 270, 14998–15004. 10.1074/jbc.270.25.14998. 7797481

[ref19] Canals A.; Purciolas M.; Aymamí J.; Coll M. The anticancer agent ellipticine unwinds DNA by intercalative binding in an orientation parallel to base pairs. Acta Crystallogr., Sect. D:Biol. Crystallogr. 2005, 61, 1009–1012. 10.1107/S0907444905015404. 15983425

[ref20] Sánchez-González Á.; Grenut P.; Gil A. Influence of conventional hydrogen bonds in the intercalation of phenanthroline derivatives with DNA: The important role of the sugar and phosphate backbone. J. Comput. Chem. 2022, 43, 804–821. 10.1002/jcc.26836. 35297513 PMC9313584

[ref21] Sánchez-González Á.; Gil A. Elucidating the intercalation of methylated 1,10-phenanthroline with DNA: the important weight of the CH/H interactions and the selectivity of CH/*π* and CH/n interactions. RSC Adv. 2021, 11, 1553–1563. 10.1039/D0RA07646E. 35424132 PMC8693566

[ref22] Gil A.; Sanchez-Gonzalez A.; Branchadell V. Unraveling the Modulation of the Activity in Drugs Based on Methylated Phenanthroline When Intercalating between DNA Base Pairs. J. Chem. Inf. Model. 2019, 59, 3989–3995. 10.1021/acs.jcim.9b00500. 31419117

[ref23] Berendsen H.; van der Spoel D.; van Drunen R. GROMACS: A message-passing parallel molecular dynamics implementation. Comput. Phys. Commun. 1995, 91, 43–56. 10.1016/0010-4655(95)00042-E.

[ref24] Hanwell M. D.; Curtis D. E.; Lonie D. C.; Vandermeersch T.; Zurek E.; Hutchison G. R. Avogadro: an advanced semantic chemical editor, visualization, and analysis platform. J. Cheminf. 2012, 4, 17 10.1186/1758-2946-4-17. PMC3542060 22889332

[ref25] Chemical Computing Group ULC Molecular Operating Environment (MOE), 2022.02. Chemical Computing Group ULC: 910–1010 Sherbrooke St. W., Montreal, QC H3A 2R7, Canada, 2023

[ref26] Case D. A.; Cheatham T. E. III; Darden T.; Gohlke H.; Luo R.; Merz K. M. Jr; Onufriev A.; Simmerling C.; Wang B.; Woods R. J. The Amber biomolecular simulation programs. J. Comput. Chem. 2005, 26, 1668–1688. 10.1002/jcc.20290. 16200636 PMC1989667

[ref27] Brooks B. R.; Brooks C. L.; Mackerell A. D.; et al. CHARMM: The biomolecular simulation program. J. Comput. Chem. 2009, 30, 1545–1614. 10.1002/jcc.21287. 19444816 PMC2810661

[ref28] Jo S.; Kim T.; Iyer V. G.; Im W. CHARMM-GUI: A web-based graphical user interface for CHARMM. J. Comput. Chem. 2008, 29, 1859–1865. 10.1002/jcc.20945. 18351591

[ref29] Huang J.; MacKerell A. D. Jr CHARMM36 all-atom additive protein force field: Validation based on comparison to NMR data. J. Comput. Chem. 2013, 34, 2135–2145. 10.1002/jcc.23354. 23832629 PMC3800559

[ref30] Mark P.; Nilsson L. Structure and Dynamics of the TIP3P, SPC, and SPC/E Water Models at 298 K. J. Phys. Chem. A 2001, 105, 9954–9960. 10.1021/jp003020w.

[ref31] Kuntworbe N.; Alany R. G.; Brimble M.; Al-Kassas R. Determination of pKa and forced degradation of the indoloquinoline antimalarial compound cryptolepine hydrochloride. Pharm. Dev. Technol. 2013, 18, 866–876. 10.3109/10837450.2012.668554. 22436019

[ref32] Torrie G.; Valleau J. Nonphysical sampling distributions in Monte Carlo free-energy estimation: Umbrella sampling. J. Comput. Phys. 1977, 23, 187–199. 10.1016/0021-9991(77)90121-8.

[ref33] Chipot C. In Free Energy Calculations. 2007.

[ref34] Ren J.; Chaires J. B. Sequence and Structural Selectivity of Nucleic Acid Binding Ligands. Biochemistry 1999, 38, 16067–16075. 10.1021/bi992070s. 10587429

[ref35] Khaliullin R. Z.; Cobar E. A.; Lochan R. C.; Bell A. T.; Head-Gordon M. Unravelling the origin of intermolecular interactions using absolutely localized molecular orbitals. J. Phys. Chem. A 2007, 111, 8753–8765. 10.1021/jp073685z. 17655284

[ref36] Aprà E.; Bylaska E. J.; De Jong W. A.; Govind N.; Kowalski K.; Straatsma T. P.; Valiev M.; van Dam H. J.; Alexeev Y.; Anchell J.; et al. NWChem: Past, present, and future. J. Chem. Phys. 2020, 152, 184102 10.1063/5.0004997. 32414274

[ref37] Epifanovsky E.; Gilbert A. T.; Feng X.; Lee J.; Mao Y.; Mardirossian N.; Pokhilko P.; White A. F.; Coons M. P.; Dempwolff A. L.; et al. Software for the frontiers of quantum chemistry: An overview of developments in the Q-Chem 5 package. J. Chem. Phys. 2021, 155, 084801 10.1063/5.0055522. 34470363 PMC9984241

[ref38] Lee C.; Yang W.; Parr R. G. Development of the Colle-Salvetti correlation-energy formula into a functional of the electron density. Phys. Rev. B 1988, 37, 785 10.1103/PhysRevB.37.785. 9944570

[ref39] Grimme S.; Antony J.; Ehrlich S.; Krieg H. A consistent and accurate ab initio parametrization of density functional dispersion correction (DFT-D) for the 94 elements H-Pu. J. Chem. Phys. 2010, 132, 154104 10.1063/1.3382344. 20423165

[ref40] Klamt A. The COSMO and COSMO-RS solvation models. Wiley Interdiscip. Rev.: Comput. Mol. Sci. 2018, 8, e1338 10.1002/wcms.1338.

[ref41] Marenich A. V.; Cramer C. J.; Truhlar D. G. Universal solvation model based on solute electron density and on a continuum model of the solvent defined by the bulk dielectric constant and atomic surface tensions. J. Phys. Chem. B 2009, 113, 6378–6396. 10.1021/jp810292n. 19366259

[ref42] Gil A.; Branchadell V.; Calhorda M. J. A theoretical study of methylation and CH/*π* interactions in DNA intercalation: methylated 1,10-phenanthroline in adenine-thymine base pairs. RSC Adv. 2016, 6, 85891–85902. 10.1039/C6RA15495F.

[ref43] Sánchez-González Á.; Castro T. G.; Melle-Franco M.; Gil A. From groove binding to intercalation: unravelling the weak interactions and other factors modulating the modes of interaction between methylated phenanthroline-based drugs and duplex DNA. Phys. Chem. Chem. Phys. 2021, 23, 26680–26695. 10.1039/D1CP04529F. 34825685

[ref44] Elleuchi S.; de Luzuriaga I. O.; Sanchez-Gonzalez A.; Lopez X.; Jarraya K.; Calhorda M. J.; Gil A. Computational Studies on the Binding Preferences of Molybdenum (II) Phenanthroline Complexes with Duplex DNA. The Important Role of the Ancillary Ligands. Inorg. Chem. 2020, 59, 12711–12721. 10.1021/acs.inorgchem.0c01793. 32806012

[ref45] Mato-López L.; Sar-Rañó A.; Fernández M. R.; Díaz-Prado M. L.; Gil A.; Sánchez-González Á.; Fernández-Bertólez N.; Méndez J.; Valdiglesias V.; Avecilla F. Relationship between structure and cytotoxicity of vanadium and molybdenum complexes with pyridoxal derived ligands. J. Inorg. Biochem. 2022, 235, 111937 10.1016/j.jinorgbio.2022.111937. 35870443

[ref46] Oliveira L. B. A.; Colherinhas G. Can CHARMM36 atomic charges described correctly the interaction between amino acid and water molecules by molecular dynamics simulations?. J. Mol. Liq. 2020, 317, 113919 10.1016/j.molliq.2020.113919.

[ref47] Ong E. E.; Liow J.-L. The temperature-dependent structure, hydrogen bonding and other related dynamic properties of the standard TIP3P and CHARMM-modified TIP3P water models. Fluid Phase Equilib. 2019, 481, 55–65. 10.1016/j.fluid.2018.10.016.

[ref48] da Fonseca A. M.; Caluaco B. J.; Madureira J. M. C.; Cabongo S. Q.; Gaieta E. M.; Djata F.; Colares R. P.; Neto M. M.; Fernandes C. F. C.; Marinho G. S.; et al. Screening of potential inhibitors targeting the main protease structure of SARS-CoV-2 via molecular docking, and approach with molecular dynamics, RMSD, RMSF, H-bond, SASA and MMGBSA. Mol. Biotechnol. 2024, 66, 1919–1933. 10.1007/s12033-023-00831-x. 37490200

