# Supplementary material for: Computational Insight into the Intercalating Properties of Cryptolepine
Source: ACS Omega. 2025 Apr 28;10(18):18283–90. doi: 10.1021/acsomega.4c08666 (PMC12079271; doi:10.1021/acsomega.4c08666)
Supplement: Supplementary file 1 — ao4c08666_si_001.pdf [file ao4c08666_si_001.pdf]

A Computational Insight into the Intercalating Properties of Cryptolepine

George Ferguson,[*,†] Louie Slocombe,[‡] John Lisgarten,[¶] David Lisgarten,[§] Colin William Wright,[‖] Rosemary Talbert,[§] Rex A Palmer,[⊥] Brendan James Howlin,[†] and Marco Sacchi[*,†]

†*Department of Chemistry and Chemical Engineering, University of Surrey, Guildford, GU2 7XH, UK.*

‡*Beyond Center for Fundamental Concepts in Science, Arizona State University, Tempe, US 85287–0506*

¶*Department of Pharmaceutical Chemistry, Faculty of Health Sciences, University of Nairobi, P.O. Box 19676-00202, Nairobi, Kenya*

§*Biomolecular Research Group, School of Psychology and Life Sciences, Canterbury Christ Church University, North Holmes Road, Canterbury Kent CT1 1QU, UK*

‖*School of Pharmacy and Medical Sciences (Faculty of Life Sciences), University of Bradford, Richmond Rd, Bradford West Yorkshire BD7 1DP, UK*

⊥*Department of Crystallography, Biochemical Sciences, Birkbeck College, Malet St, London WC1E 7HX, UK.*

E-mail: g.a.ferguson@surrey.ac.uk; m.sacchi@surrey.ac.uk

Initial Structure

To create the initial structure, including the backbone, we optimized intercalated Cryptolepine using Density Functional Tight Binding (DFTB) via the DFTB+ code[1] on a ring model of the DNA. This includes the nearest base pairs surrounding the intercalant and the backbone connecting these two DNA chains. These backbones included hydrogen to cap off the ends at the oxygen O3' on both ends of the backbone and replicated on the other backbone to a total atom count of 163 and 165 for C-G and A-T base pairs, respectively. The optimized configuration generated by DFTB+ was then used as an input for DFT calculations. This was not performed for the GC and AT base pairs, which were directly calculated via DFT. See the GitHub for the exact QM structures in the optimization - Ring Model file. See figure 1 below for an example of the QM structures:

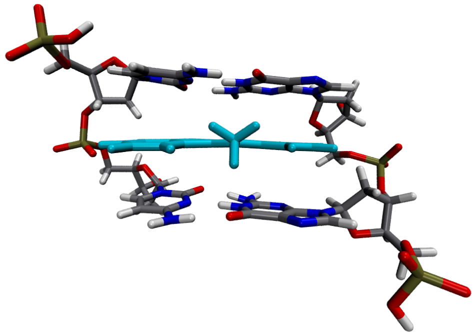

(a) CC

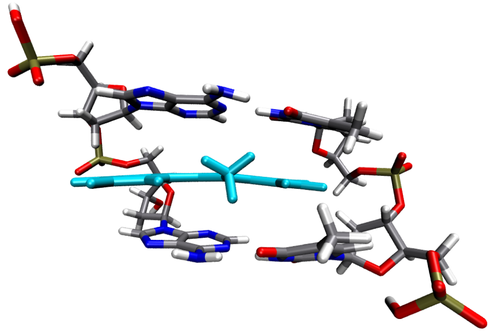

(b) AA

Figure S 1: Examples of the QM structure (CC and AA shown) used to run the QM calculations. These are a ring model of the DNA surrounding Cryptolepine (teal), and all QM structures used followed this form.

Boltzmann Probability Distribution

To find an occupation probability with the potential wells shown in Table 2, we used a Boltzmann Probability Distribution to find a distribution of Cryptolepine across various intercalant sites at thermal equilibrium:

$$P_s = \frac{\exp\big(-\beta E_s\big)}{\sum_i \exp\big(-\beta E_i\big)} \tag{Eq. S1}$$

Where P_s is the probability of state s, E_s is the energy of state s, and the denominator is the sum of all possible energy states, E_i, Cryptolepine could be in. Using this to estimate occupation, we assume Cryptolepine has time to move between and enter each possible binding site.

RMSD Calculation

We investigated the classical stability of the intercalant throughout 20 ns inside a solvated DNA chain for all base pair combinations. The system was set to a thermal bath of 310.15 K and equilibrated for an NVT simulation.

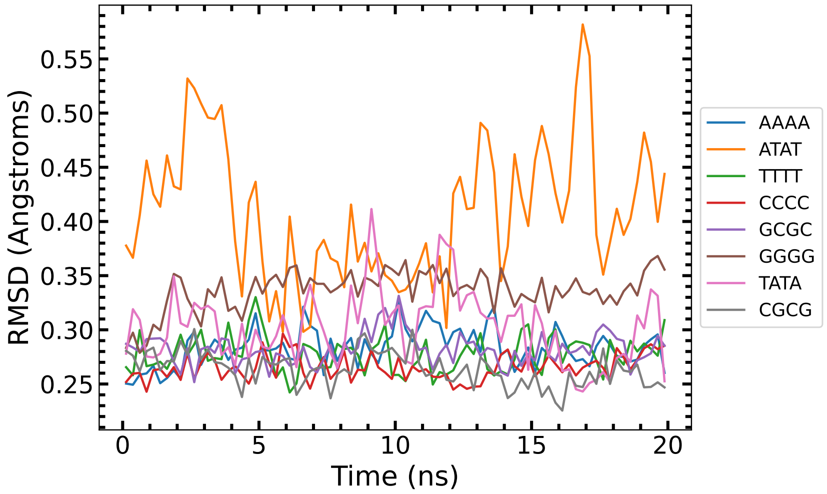

Figure S 2: Root Mean Square (RMS) Distance plot of a single Cryptolepine molecule while intercalated in the center of a 12 base DNA chain in a solvent.

The above figure demonstrates classically that Cryptolepine remains stable in all combinations of DNA. Notably, the alternating A-T base pair system is less stable than other base pair combinations; however, the RMS variation it undergoes is below 1.0 Å; thus, the Cryptolepine remains relatively stable even in this system. For all other cases, the RMS

distance between Cryptolepine and base pairs is nearly identical; therefore, no preference can be detected.

DNA Chain Stability

We used the DNA structure before the intercalant was included to ensure the structure remained stable during the simulation. We ran the MC calculation with the same parameters for 20 ns to match the longest simulation we performed. We ran four chains representing the alternating and non-alternating forms of C-G and A-T pairs. We ran each structure three times, and the RMSD and Standard Deviation of this calculation are listed in the table below:

Table S 1: RMSD average and standard deviation from MD calculation of four different DNA 12 base pair DNA chains (CC, CG, TT, TA) in the solvent over 20 ns NVT at 310.15 K. All distance values are in Å.

DNA Bases	Average RMSD	StD
CC 1	3.840	0.7844
CC 2	6.224	1.3130
CC 3	3.981	0.6841
CG 1	2.467	0.3617
CG 2	2.752	0.3467
CG 3	2.257	0.2963
TT 1	3.082	0.5824
TT 2	4.031	0.9941
TT 3	3.802	0.8284
TA 1	4.923	1.3806
TA 2	3.478	1.1280
TA 3	3.605	1.2700

MM Hydrogen Bond Distances and Angles

The number and length of all hydrogen bonds and angles taken from the energy-minimized structures are listed in the table below. Bond ID follows the nomenclature of the nucleobase, C, G, A, and T, followed by its index in the DNA chain. The O or N represents the acceptor

atom for the hydrogen bond. Cr is defined as Cryptolepine, and H represents hydrogen with its index listed after the H.

Table S 2: CC hydrogen bond distance and angles were taken from the energy-minimized FEP structures.

Bond ID	Hydrogen Bond Distance (Å)	Twist Alignment (Deg)
G18-O6-Cr-H13	2.55	113.03
G17-O6-Cr-H12	2.89	132.52
G17-N7-Cr-H12	2.81	147.35
G18-O5'-Cr-H2	3.08	150.56
G18-O4'-Cr-H2	3.24	97.56
G18-O4'-Cr-H3	2.69	113.32
C6-O4'-Cr-H6	1.98	163.13
C6-O4'-Cr-H7	3.28	115.85

Table S 3: CG hydrogen bond distance and angles were taken from the energy-minimized FEP structures.

Bond ID	Hydrogen Bond Distance (Å)	Twist Alignment (Deg)
G17-O4'-Cr-H6	2.21	138.00
G17-O4'-Cr-H7	2.58	122.75
G17-O5'-Cr-H7	2.71	166.01
G7-O4'-Cr-H4	3.23	110.73
G7-O4'-Cr-H3	2.81	125.66

Table S 4: GC hydrogen bond distance and angles were taken from the energy minimized FEP structures.

Bond ID	Hydrogen Bond Distance (Å)	Twist Alignment (Deg)
G5-O6-Cr-H12	2.98	134.07
G17-O6-Cr-H13	3.00	96.03
G17-O6-Cr-H11	2.92	100.61
C18-O4'-Cr-H6	1.91	161.20
C18-O5'-Cr-H7	3.13	151.84
C18-O2P-Cr-H7	3.13	148.62
C6-O4'-Cr-H3	2.64	121.06
C6-O4'-Cr-H2	3.21	102.23
C18-O2-Cr-H5	2.60	96.42

Table S 5: GG hydrogen bond distance and angles were taken from the energy-minimized FEP structures.

Bond ID	Hydrogen Bond Distance (Å)	Twist Alignment (Deg)
G5-N7-Cr-H12	2.86	121.69
G5-O6-Cr-H11	3.09	115.18
C18-O4'-Cr-H3	2.88	142.75
G6-O4'-Cr-H6	1.87	161.74
G6-O5'-Cr-H7	2.52	145.64
G6-O2P-Cr-H7	2.84	110.22

Table S 6: TT hydrogen bond distance and angles were taken from the energy-minimized FEP structures.

Bond ID	Hydrogen Bond Distance (Å)	Twist Alignment (Deg)
A18-N6-Cr-H11	2.67	126.43
T5-O4-Cr-H12	2.64	134.03
A17-N7-Cr-H13	3.00	138.99
A18-O4'-Cr-H3	2.57	112.86
A18-O5'-Cr-H2	3.13	145.55
T6-O4'-Cr-H6	2.00	154.91
T6-O4'-Cr-H7	2.99	118.86
T6-O5'-Cr-H7	2.82	174.14

Table S 7: TA hydrogen bond distance and angles taken from the energy-minimized FEP structures.

Bond ID	Hydrogen Bond Distance (Å)	Twist Alignment (Deg)
T5-O4-Cr-H12	2.53	117.86
A18-O4'-Cr-H3	2.77	126.52
A18-O5'-Cr-H3	3.14	130.78
A18-OP2-Cr-H2	2.52	163.50
A6-O4'-Cr-H6	2.02	145.19
A6-O4'-Cr-H7	2.62	124.58
A6-O5'-Cr-H7	2.99	164.37

Table S 8: AT hydrogen bond distance and angles taken from the energy-minimized FEP structures.

Bond ID	Hydrogen Bond Distance (Å)	Twist Alignment (Deg)
T18-O4'-Cr-H6	2.14	143.26
T18-O4'-Cr-H7	2.69	125.01
T18-O5'-Cr-H7	2.72	166.22
T18-OP2-Cr-H8	2.62	131.44
T6-O4'-Cr-H3	2.86	122.76

Table S 9: AA hydrogen bond distance and angles taken from the energy-minimized FEP structures.

Bond ID	Hydrogen Bond Distance (Å)	Twist Alignment (Deg)
T5-O4-Cr-H13	3.23	133.62
A18-O4'-Cr-H7	2.70	111.66
A18-N4'-Cr-H8	2.65	112.64
A18-O5'-Cr-H8	3.10	163.21
T6-O4'-Cr-H5	2.38	143.41
T6-O4'-Cr-H4	2.62	131.24
T6-O5'-Cr-H4	2.91	153.13
T6-OP2-Cr-H3	2.96	142.13

QM Hydrogen Bond Distances and Angles

The number and length of all hydrogen bonds and angles were taken from the force-optimized QM ring models of DNA via NWChem. Bond ID follows the index of the QM structure (see GitHub for the .xyz file of the structure), with the name of the acceptor atom, O or N, followed by its index value. The hydrogen is then listed, followed by its index value.

Table S 10: CC hydrogen bond distance and angles taken from the optimized ring model.

Bond ID	Hydrogen Bond Distance (Å)	Twist Alignment (Deg)
O16-H162	2.66	130.93
O38-H160	3.10	107.09
O28-H153	2.71	113.38
O28-H152	2.74	112.80
O25-H152	2.33	168.81
O88-H156	2.98	142.18
O84-H156	2.63	120.33
O84-H157	2.62	121.60

Table S 11: CG hydrogen bond distance and angles taken from the optimized ring model.

Bond ID	Hydrogen Bond Distance (Å)	Twist Alignment (Deg)
O38-H160	2.90	122.05
O16-H162	2.94	126.78
O25-H152	2.60	166.81
O28-H152	2.70	120.09
O28-H153	3.05	107.79
O88-H155	2.22	135.64
O88-H156	2.76	117.24
O85-H156	2.35	168.76
O84-H157	2.49	135.40

Table S 12: GC hydrogen bond distance and angles taken from the optimized ring model.

Bond ID	Hydrogen Bond Distance (Å)	Twist Alignment (Deg)
O18-H160	2.75	128.68
O41-H153	2.28	165.42
O41-H154	3.25	132.74
O38-H152	2.75	114.44
O38-H153	2.67	116.99
O37-H152	2.79	157.49
O114-H155	2.78	112.13
O107-H155	3.29	124.88
O107-H156	2.30	158.27
O104-H156	2.63	129.48
O104-H157	3.15	110.59
O103-H157	3.15	157.53

Table S 13: GG hydrogen bond distance and angles taken from the optimized ring model.

Bond ID	Hydrogen Bond Distance (Å)	Twist Alignment (Deg)
O17-H156	2.99	119.03
O50-H157	3.22	149.40
O40-H152	2.59	120.30
O40-H153	2.90	110.10
O37-H153	2.26	177.80
O100-H149	2.64	144.82
O101-H150	2.51	143.45
O101-H149	2.40	148.45
O111-H151	3.16	124.11

Table S 14: TT hydrogen bond distance and angles taken from the optimized ring model.

Bond ID	Hydrogen Bond Distance (Å)	Twist Alignment (Deg)
O128-H163	2.48	129.15
O14-H154	2.40	173.67
O17-H154	2.67	115.56
O17-H155	2.76	112.43
O46-H159	2.44	130.13
O46-H158	2.79	114.23
O47-H158	2.44	173.39
O50-H157	2.18	135.79
O50-H158	2.74	118.26

Table S 15: TA hydrogen bond distance and angles taken from the optimized ring model.

Bond ID	Hydrogen Bond Distance (Å)	Twist Alignment (Deg)
O114-H164	2.89	145.80
O128-H163	2.55	119.82
O14-H154	2.49	172.62
O17-H154	2.74	117.09
O17-H155	2.92	110.63
O46-H159	2.53	129.15
O46-H158	2.81	115.90
O47-H158	2.36	174.94
O50-H158	2.88	116.85
O50-H157	2.14	140.98

Table S 16: AT hydrogen bond distance and angles taken from the optimized ring model.

Bond ID	Hydrogen Bond Distance (Å)	Twist Alignment (Deg)
O40-H155	3.05	112.08
O40-H154	2.72	124.63
O37-H154	2.73	168.28
O107-H157	1.90	157.12
O107-H158	2.83	138.14
O104-H158	2.59	128.08
O103-H158	2.79	150.30

Table S 17: AA hydrogen bond distance and angles taken from the optimized ring model.

Bond ID	Hydrogen Bond Distance (Å)	Twist Alignment (Deg)
O114-H160	2.65	151.70
O82-H159	2.43	145.70
O35-H154	2.93	153.96
O36-H154	2.57	127.14
O36-H153	2.88	142.37
O39-H153	1.88	159.16
O39-H152	3.17	114.67
O104-H150	2.56	125.02
O101-H150	2.90	138.27

References

(1) Hourahine, B. et al. DFTB+, a software package for efficient approximate density functional theory based atomistic simulations. *The Journal of Chemical Physics* **2020**, *152*, 124101.
